# Sol-Gel Chemistry: From Molecule to Functional Materials

**DOI:** 10.3390/molecules25112538

**Published:** 2020-05-29

**Authors:** Sébastien Clément, Ahmad Mehdi

**Affiliations:** ICGM, Univ Montpellier, CNRS, ENSCM, CC1701, Place Eugène Bataillon, CEDEX 05, F-34095 Montpellier, France

**Keywords:** sol-gel, hydrolytic, non-hydrolytic, self-assembly, catalysis, titanium dioxide, PAHs adsorption, dye-sensitized solar cells (DSSCs), catalysis, anticorrosion, antifouling, coatings

Through this Special Issue, you will discover the potentiality of inorganic polymerization (sol-gel process) which is a unique and versatile way for the preparation of materials. Indeed, this process can design (à la carte) an oxide or a functional hybrid material while controlling the structure, texture, shape, and its physicochemical properties. The strength of this process lies in the choice of the molecular precursor (elementary building block) and the conditions under which the materials are produced. These monitor the nanoscale interactions between the bricks which become the driving force for an ideal assembly of the final material. We have grouped 21 articles covering a broad aspect of this chemistry with applications crossing several domains ranging from chemical (catalysis, …) to biological (imaging, …) through to physical (optical, …) properties.

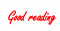

## 1. Synthetic Methodology/Texture/Structuration

Britt et al. presented a biocompatible approach for the preparation of the confinement of biomolecules in a silica matrix [1]. This synthesis, assisted by microwave, allowed preparing homogenous nanocomposites in terms of composition and surface functional groups distribution. They used a spider silk protein as the starting biomacromolecule and silylated precursors under well-chosen sol-gel conditions which gave rise to submicron spherical particles of silk@ORMOSIL. It was shown by ATR-FTIR spectroscopy that the organosilane blocks played a crucial role in the assembly process of protein in the nanocomposites. In addition, methods to tune the secondary structures, and in particular, β-sheets which are the cross-linking moieties in spider silks and other self-assembling fibrillar proteins, provided a unique means to promote protein interactions and, subsequently, epitaxial growth and biomineralization could be tuned for user-defined applications of the protein@silica nanocomposites.

The same authors described an optical approach to follow the kinetic of the polycondensation process of organosilanes [2]. Instead of ^29^Si NMR spectroscopy, which is commonly used, they turned to original and quick methods such as *in situ* optical turbidity scanning and dynamic light scattering. For their study, two organosilanes were chosen, *n*-propyltrimethoxysilane (nPM) and its trifluorinated analog, 3,3,3-trifluoropropyltrimethoxysilane (3F), and their hydrolysis and polycondensation were investigated at different acidic pH levels. Thanks to the complete insolubility of these precursors, and therefore their clearly observed phase separation in water, it was easy to follow the reaction kinetics using a Turbiscan instrument to monitor the hydrolysis step through solubilization of the neat silane lens, while simultaneously tracking condensation-induced turbidity throughout the bulk solution. Dynamic light scattering confirmed the silane condensation step and particle aggregation processes were reported by the turbidity scanning. Since the hydrolysis and polycondensation were carried out under acidic conditions, it was observed that the organosilane nPM remained on the surface, whereas, for the organosilane 3F, the condensation produced aggregates that settled to the bottom of the tube. This observation can be explained by the destabilization of siliconium positive charged intermediate species (SiOH_2_^+^, SiOHMe^+^, etc.) due to the electron-withdrawing character of the CF_3_-groups.

Large size silica aerogels with good thermal insulating properties were prepared using the sol-gel process in aqueous media by Shen et al. [3]. In their paper, the authors described an easy and inexpensive synthesis of silica monoliths, as well as their textural characterization and thermal properties. Starting from colloidal solution silica, by changing the pH, the hydrolysis and polycondensation steps could be effectively monitored. The polymerization was assisted by cetyltrimethylamonium bromide (CTAB) under basic media, thanks to the ionic interaction between the cationic head and anionic species of silica source in the presence of different amounts of non-ionic templating agents (polyethylene oxide PEG400). They showed that a small amount of PEG400, which is the monolith that is obtained after thermal treatment at 600 °C, has interesting textural properties (230 m^2^/g as surface area and 15 nm of pore size). More interestingly, its low density of 221 Kg·m^−3^, low thermal conductivity of 0.0428 W·m^−1^·K^−1^, and scalability opened a real opportunity for a promising industrial application as thermal insulation materials in several domains.

In addition, the potentiality of inorganic polymerization for the design of multifunctional silica was shown in the review by Abdel-Rehim et al. [4] using imprinted polymers (MIPs) as advantaged materials for selective sorption, thanks to multiple recognition sites for a template molecule or nano-objects of interest based on their size, structure, and pendant functional groups. Since the pioneering work of Mosbach and coworkers [5,6], a large number of papers that have considered organic MIPs have been reported. However, the work in these papers obviously suffered from thermal treatment (degradation at >150 °C) and mechanical stability issues, unstable texture, and poor capacity. In their review, Abdel-Rehim et al. summarized the different silica-based molecularly imprinted polymers (MIPs) prepared by the sol-gel approach with improved thermal and chemical stability thanks to an inorganic matrix [4]. The combination of sol-gel and molecularly imprinted tools has given rise to powerful and tunable separation material with high selectivity and high loading capacity, opening an appealing perspective for future applications in the analytical and bioanalytical fields.

Finally, bioglass-based zinc-doped hydroxyapatite (HAp) obtained using the sol-gel method were described by Predoi et al. [7]. In their work, the authors described the synthesis and characterization of regularly dispersed zinc ions in the hydroxyapatite matrix by hydrolysis and polycondensation of Ca(NO_3_)_2_·4H_2_O, (NH_4_)_2_HPO_4_, and Zn(NO_3_)_2_·6H_2_O. The doping was confirmed and quantified by XPS analysis and thanks to XRD and Rietveld analysis, the authors revealed that Zn@HAp (0.7% of Zn and 99.3% of Ca) had the same structure (P63/m spatial group) as Zn-free hydroxyapatite. They proposed a model to verify the stability of HAp and Zn@HAp gels by ultrasound measurements using the finite elements method (FEM) to simulate the wave propagation. The proposed model demonstrated that the nondestructive ultrasonic method was suitable to characterize undiluted bioglass gels.

## 2. Optical Properties

Mehdi, Clément et al. showed that optical properties of polythiophene embedded in a silica matrix could be controlled by polymerizing of the inorganic part of mono-silylated-thiophene precursor [8]. The crosslinking in water was assisted by self-assembly through van der Waals interactions of alkylene chains, π-stacking interactions between the thiophene units, and hydrogen bonding interactions between ureido groups. This supramolecular approach based on multiple noncovalent interactions also allowed the formation of nanostructured layered conjugated polymers-based hybrid materials.

Deng et al. reported the hydrophobic treatment of potassium dihydrogen phosphate (KDP) crystal by surface coating with trimethysilyl-functionalized silica nanoparticles (Nps) for high-power laser systems [9]. By controlling the embedding ratio of hydrophobic nanoparticles, they showed that the refractive index of such coatings could be tuned arbitrarily in the range of 1.21–1.44, which endowed the KDP optical component with exceptional transmission capability, as well as the moisture proof effect. In addition, transmittance of such a dual-layer coating system could reach 99.60% and 99.62% at 1064 and 532 nm, respectively, by precisely matching the refractive index of both layers. Finally, the authors showed the good stability (six months) of the thin hydrophobic layer at a high humidity ambient condition of 80% RH.

Optical fibers containing rare-earth doped nanoparticles have been investigated to develop new devices such as fiber lasers or amplifiers [10]. Thanks to this route, alteration of the spectroscopic properties of RE ions have already been reported [11]. However, the broad size distribution of vitreous nanoparticles does not discriminate between the role of their composition and their size on the luminescent properties, as already reported for crystalline nanoparticles. In this context, Mehdi et al. described the europium and magnesium ions confinement in silica nanoparticles and their luminescent properties before and after annealing at 900 °C [12]. The sol-gel process remains as one of the most important approaches for the preparation of such nanoparticles with a diameter larger than 50 nm using the Stöber method. Smaller nanoparticles (around 10 nm,) can also be obtained by sol-gel in reverse micro emulsion method (water in oil) in which hydrolysis and the polycondensation take place in the hydrophilic micelles that play the role of nanoreactors. Thus, silica nanoparticles with several molar contents of europium (0.2%, 0.5%, and 1%) were prepared in one step. In particular, it was shown that the addition of magnesium ions modified the glass structure and the thermal treatment eliminated the aqueous environment, modifying the structure ordering. The emission spectra and the decay time curves clearly showed the advantages of the Mg addition and the annealing on the photoluminescent properties.

## 3. Health and Biomedical Applications

Durand et al. reported the synthesis and characterization of framework-functionalized mesoporous silica nanoparticles with the aim of CO_2_ release which acted as a contrast agent for high intensity focused ultrasounds (HIFU) [13]. In this study, several multi-silylated precursors containing *tert*-butoxycarbonyl (BOC) group were synthesized. The BOC group was judiciously chosen for its ability to release CO_2_ in water at 90–100 °C. Several multifunctional mesoporous nanoparticles with controlled size and pore diameter were prepared by sol-gel in the presence of CTAB as a structure directing agent. Interestingly, total survival of the BOC group during the inorganic polymerization process was observed and even after thermal treatment at 120 °C. CO_2_ release was observed at pH to 1, at 100 °C but, unfortunately, all BOC groups remained intact at pH 5.5 (conditions of HIFU). Despite the observed results, these nanoparticles are a good example of the design of nanomaterials in which molecular chemistry has a crucial role to play in the future.

In the last decade, functional nanoparticles designed for imaging and therapeutic applications were of great interest for nanomedicine domain and remain a great challenge [14,15,16,17]. The work presented by Charnay et al. is part of this exiting research [18]. For this study, the authors described the preparation of two different upconverting-based hybrid nanoparticles by hydrolysis and polycondensation of bis(triethoxysilyl)ethylene and bis(triethoxysilyl)ethane in the presence of cetyltrimethylammonium bromide (CTAB) as the templating agent. Since functional PMOs Nps have been shown to be very efficient for drug delivery, and upconverting nano-tools are interesting for near-infrared and X-ray computed tomography imaging, depending on the used matrix, the authors decided to make a tunable doping of their nanoparticles with Yb^3+^-Er^3+^ as the upconverting system and NaYF_4_ or BaLuF_5_ as the matrix. They clearly showed that, in the case of bis(triethoxysilyl)ethane-based nanoparticles, upconversion nanocrystals were well confined, giving rise to interesting features for theranostics applications such as imaging upon near-infrared or X-ray excitation respectively.

Biomaterial based on polyethylene glycol (PEG)@silica composite and doped chlorogenic acid (CGA) has been reported by Catauro et al. [19]. In fact, CGA is an attractive biomolecule due to its potential use as a preventive and therapeutic agent in many diseases, as well as cancer. The sol-gel process is perfectly compatible with biomolecules, since it can be operated under fully biocompatible conditions. Materials with different amounts of CGA were characterized by usual techniques, i.e., their bioactivity and ability to produce a hydroxyapatite layer on their surface upon incubation with simulated body fluid (SBF) was demonstrated and evaluated by scanning electron microscopy. More interestingly, the authors showed that these nanocomposites (SiO_2_/PEG/CGA) displayed clear antiproliferative effects in different tumors, including breast cancer and osteosarcoma cell lines in a CGA dependent manner, but not in normal cells. These results highlight the interest of CGA as a possible anticancer agent and illustrate the potential for clinical applications of such biomaterials.

## 4. (Bio)catalysis Applications

Developing sol-gel-derived catalysts (entrapped organic molecules, metal complexes, or metal nanoparticles, etc.) have been the subject of intense research since the late 1980s [20,21] and still generate ongoing interest as illustrated by the contributions of Zaharescu et al. [22] and Luque et al. [23] who both described how lipase could be immobilized by the sol-gel method. Zaharescu et al. described the immobilization of lipase from *Rhizopus orizae* in spherical SiO_2_ and tubular SiO_2_ (two diameters were investigated, 200–300 nm and 20–50 nm) [22]. This immobilization was performed at pH = 6, ranging between the isoelectric point of silica and lipase. Finally, the lipase enzymatic activity immobilized from SiO_2_ was determined by studying the hydrolysis of *p*-nitrophenyl acetate hydrolysis revealing that the highest activity was obtained for the thinner tubular SiO_2_.

Luque et al. investigated the effect of sol-gel immobilization on the esterification reaction of valeric acid [23]. After optimization of the reaction conditions (biocatalyst load, presence of water, reaction mixture, etc.), the conversion of valeric acid into ethyl valerate was investigated under conventional and microwave heating leading to 80%–85% yield range. Enzyme denaturalization probably occurred for microwave heating while the immobilized enzyme remained stable under conventional heating, making this procedure implementable in many industrial fields.

Exploiting the superacidity of a sulfated zirconia (SO_4_^2-^/ZrO_2_) for catalytic hydrogenolysis, Ren et al. described the depolymerization of larix bark polymeric proanthocyanidins (LPPC) into larix bark oligomeric proanthocyanidins (LOPC) leading to a depolymerization yield of 54% [24]. The obtained LOPC showed good antioxidant properties and fluorescent properties sensitive to pH, solvent polarity, and metal ions which allowed its use for fluorescent labeling in biological studies.

## 5. Anticorrosion and Antifouling Coatings

The versatile sol-gel process also offers the opportunity to develop multifunctional hybrid coatings that can ensure an efficient barrier effect against aggressive environments [25,26]. In this respect, Ansart et al. studied the adhesion properties and the corrosion resistance of phosphated zinc substrates treated with a hybrid (organic-inorganic) sol-gel [27]. Different formulations based on 3-glycidoxypropyltrimethoxysilane (GPTMS)/aluminum-tri-sec-butoxide (ASB) and 3-(trimethoxysilyl)propylmethacrylate (MAP)/tetraethylorthosilicate (TEOS) were tested. To determine the most suitable formulation, surface mechanical investigations, topographic analyses, salt-spray tests, and microstructural characterizations were performed. On the basis of these results, the TEOS/MAP-based coating was found to be the best in terms of adhesion performances (top-coat paint, microscratch, and shock) and corrosion resistance. Further improvements could be achieved by tuning the TEOS/MAP formulation with the addition of (1-[3-(trimethoxysilyl)propyl]ureido (UPS); the best results being obtained for a TEOS-MAP sol-gel system in which 50% of MAP precursor is replaced by UPS. Overall, these results showed that the sol-gel network through the presence of strong covalent bonds can improve the adhesion properties, as well as the barrier effect, to protect the substrate against corrosion.

In a second contribution, the same group described how it was possible to improve the aluminum alloy corrosion resistance with an organic-inorganic hybrid (OIH) sol-gel coating elaborated from glycidoxypropyltrimethoxysilane (GPTMS), zirconium (IV) propoxide (TPOZ), and aluminum tri-sec-butoxide (ASB) precursors [28]. Very good corrosion resistance (higher than 500 h) was notably achieved with this highly condensed hybrid sol-gel coating of thickness lower than 4 mm. Such performance was assigned to the formation of an aluminosilicate matrix reinforced by zirconium for the highly condensed inorganic part and the presence of the cerium (III) nitrate hexahydrate precursor acting as a corrosion inhibitor.

In addition to corrosion, sol-gel derived coatings are also attractive candidates for both preventing biofouling and enhancing the hydrodynamic properties of boat and ship hulls [29,30]. The study by Regan et al. illustrated this purpose with the aim of determining the antifouling effectiveness of five novel silica-based coatings possessing different hydrophobicities, wettability characteristics, and roughness which are deployed in the marine environment [31]. The study was based on the evaluation of primary fouling (at nine months), and then macrofouling (4 months later) on these samples. From these results, it appeared that hybrid sol-gel coatings, in particular HC006 that showed the greatest diversity of organism colonization in the initial stages, reduced the likelihood of permanent biofilm. In the case of macrofouling, DMDEOS was found to be the most effective with good robustness allowing its use in dynamic conditions.

## 6. TiO_2_-Based Hybrid Materials

In addition to the silica-based hybrid materials, TiO_2_-based nanomaterials have been attracting tremendous interest due to their wide range of applications such as photocatalysis, catalysis, sensing, and energy conversion [32]. Mutin et al. described the preparation of mesoporous TiO_2_, investigating how the texture and morphology could be tuned by changing the synthetic route or the nature of the solvent [33]. Three non-hydrolytic sol-gel routes based on the reaction of TiCl_4_ with iPr_2_O and Ti(OiPr)_4_ with stoichiometric amounts of acetophenone or benzoic anhydride were performed leading to TiO_2_ rounded nanoparticles, platelets, or nanorods with different porosities.

Functionalization of titanium oxide with a series of organophosphorus molecules (R–OPO_2_H_2_) holding different unsaturated groups (R = vinyl, phenyl, and naphtyl) and spacers was described by Daniele et al. [34]. Then, these hybrid materials were exploited as nanoadsorbants for the adsorption of polycyclic aromatic hydrocarbons (PAHs) through π–π stacking interactions. They exhibited high adsorption capacities (1 mg.mL^−1^) and tunable selectivity, depending on the nature of the unsaturated-based phosphonate ligands, on the self-agglomeration of the surface-functionalized nanoparticles, and on the textures of the nanomaterials.

The photovoltaic properties of chlorophyll-a derivatives grafted to a mesoporous TiO_2_ electrode were investigated by Clément et al. in “all solid-state” dye sensitized solar cells (DSSCs) using 2′,2′,7,7′-tetrakis[*N*,*N*-di(4-methoxyphenyl)amino]-9,9′-spirobifluorene as the hole transport material [35]. In depth analysis of the parameters (structure, nature of the anchoring group, adsorption, etc.) and their relationships with the PCEs by combining density functional theory (DFT) calculations, optical and photovoltaic studies, and electron paramagnetic resonance analysis revealed that the recombination kinetics, the frontier molecular orbitals of these dyes, and the adsorption efficiency onto the TiO_2_ surface are key parameters.

The optical properties of TiO_2_ were also exploited by Gonçalves et al. in their study of two new TiO_2_-based amorphous TiO_2_ (undoped and N-doped) films [36]. The bactericidal potential (even in the absence of daylight and for only 1 h of incubation period) were investigated using their ability to react with oxygen-containing molecules and form reactive oxidant species (ROS), which in turn disrupted the cellular membrane of simple biological systems, such as bacteria. Studies on bactericide properties on *Escherichia coli* strain revealed a bactericidal efficiency above 50%, which seems to be promising for their use in environmental and medical applications.
